# DDX58 expression promotes inflammation and growth arrest in Sertoli cells by stabilizing p65 mRNA in patients with Sertoli cell-only syndrome

**DOI:** 10.3389/fimmu.2023.1135753

**Published:** 2023-03-22

**Authors:** Hao Sun, Zhan Yang, Zhihai Teng, Yanping Zhang, Zhenwei Han, Chao Xu, Zhu Wang, Hu Wang, Hongzhuang Wen, Xiaodong Chen, Changbao Qu, Yaxuan Wang

**Affiliations:** ^1^ Department of Urology, The Second Hospital of Hebei Medical University, Shijiazhuang, China; ^2^ Molecular Biology Laboratory, Talent and Academic Exchange Center, The Second Hospital of Hebei Medical University, Shijiazhuang, China

**Keywords:** sertoli cell-only syndrome, sertoli cell, DDX58, p65, inflammation

## Abstract

Sertoli cell -only syndrome (SCOS) is a type of testicular pathological failure that causes male infertility and no effective treatment strategy, is available for this condition. Moreover, the molecular mechanism underlying its development remains unknown. We identified DExD/H-Box helicase 58 (*DDX58*) as a key gene in SCOS based on four datasets of testicular tissue samples obtained from the Gene Expression Synthesis database. DDX58 was significantly upregulated in SCOS testicular Sertoli cells. Moreover, high expression of DDX58 was positively correlated with the expression of several testicular inflammatory factors, such as IL -1β, IL-18, and IL-6. Interestingly, DDX58 could be induced in the D-galactose (D-gal)-stimulated TM4 cell injury model. Whereas silencing of DDX58 inhibited D-gal -mediated p65 expression, inflammatory cytokine release, and growth arrest. Mechanistically, we found that DDX58 acts as an RNA-binding protein, which enhances p65 expression by promoting mRNA stability. Furthermore, p65 gene silencing decreased the expression of inflammatory cytokines and inhibition of cell growth in D-gal-induced cells. In conclusion, our findings demonstrate that DDX58 promotes inflammatory responses and growth arrest in SCOS Sertoli cells by stabilizing p65 mRNA. Accordingly, the DDX58/p65 regulatory axis might be a therapeutic target for SCOS.

## 1 Introduction

Sertoli cell -only syndrome (SCOS) is a type of pathological testicular failure that causes male infertility and is characterized by azoospermia, high levels of serum follicle-stimulating hormone, and loss of spermatogenic cells in the testis, leaving only Sertoli cells (1–3). Approximately 50% of infertility cases are caused by male factors (2), and failure of sperm production is the most serious cause of male infertility, with approximately 10%–15% of cases presenting with azoospermia (3). The occurrence and development of SCOS are related to various factors, including radiotherapy and chemotherapy (4, 5), cryptorchidism (6), inflammatory response (7), and chromosomal abnormalities (8, 9). Dysfunction of Sertoli cells, an essential component of the blood–testis barrier, is considered an important cause of SCOS (4, 10, 11). Numerous mutations in Sertoli cells and aberrant expression of genes encoding PIWIL2, FANCM, PLK4, TEN1, ZMYND15, and HSF2, affect spermatogenesis (12–15). However, the specific etiology and pathogenesis of SCOS remain unclear. Further, there are no specific drugs or treatments for SCOS (1). Thus, to meet the diagnostic and therapeutic needs of patients with SCOS, it is essential to explore its molecular causal factors.

DExD/H-Box helicase 58 (*DDX58*) encodes retinoic acid-inducible gene-I (RIG-I). DDX58, a receptor which was first identified in the innate immune response recognizes cytoplasmic viral nucleic acids and activates downstream signaling cascades, leading to the production of type I interferon and proinflammatory cytokines (16). In addition, DDX58 participates in various physiological processes (17), including viral invasion (18), tumor immune response, inflammatory response, autophagy, and apoptosis, as well as classic Singleton–Merten syndrome (19–21). For example, DDX58 is associated with susceptibility to severe influenza virus infection in adolescents (22). Moreover, Xian et al. found that leucine-rich repeat containing protein 59(LRRC59) regulates type I interferon signaling by inhibiting p62-mediated DDX58 autophagy (23). The MAPK pathway inhibits inflammatory reprogramming and sensitizes tumors to the targeted activation of DDX58 (24). In addition, several studies have shown that DDX58 closely correlates with the occurrence and progress of classic Singleton—Merten syndrome (25, 26). However, to the best of our knowledge, the role of DDX58 in SCOS has remained largely unexplored.

In this study, we revealed that DDX58 expression was upregulated in SCOS Sertoli cells using bioinformatics analysis. Our results showed that DDX58 expression correlates with the inflammatory environment in the testis. In addition, DDX58, a RNA-binding protein, promotes inflammatory responses and growth arrest in Sertoli cells by stabilizing the mRNA of the nuclear transcription factor p65. This newly discovered regulatory axis might be a therapeutic target for SCOS.

## 2 Material and methods

### 2.1 Data acquisition

Microarray data from testicular tissues of patients with SCOS or obstructive azoospermia (OA) were obtained from the Gene Expression Synthesis (GEO)(https://www.ncbi.nlm.nih.gov/geo/) database. The mRNA expression profiling datasets GSE6023 (27), GSE4797 (28), GSE45885 (29), and GSE45887 (30) were downloaded in data format MINiML. **Table 1** provides information about the four datasets.

**Table 1:** Details of GEO SCOS date.

GEO accession	Platform	Source tissue	Samples		Gene
			NS	SCOS	
GSE6023	GPL2891	Testis	1	5	mRNA
GSE4797	GPL2891	Testis	12	5	mRNA
GSE45885	GPL6244	Testis	4	7	mRNA
GSE45887	GPL6244	Testis	4	5	mRNA

GEO, Gene Expression Omnibus; NS, Normal Spermatogenesis; SCOS, Sertoli Cell-only Syndrome.

### 2.2 Data collection, normalization, and identification of differentially expressed genes

Sample data from the same platform were merged. Based on the affy package of R software (version 4.0.3) (https://www.bioconductor.org/), the combined datasets were preprocessed and normalized *via* the robust multiarray average method. Genes with an adjusted p -value of <0.05 and absolute fold change of ≥1 were considered as differentially expressed genes (DEGs) using the ‘limma’ package of R software (31). Box line plots, and volcano maps were constructed using ‘ggplot2’ package (version 3.3.2)(https://www.rdocumentation.org/packages/ggplot2/versions/3.3.2).

### 2.3 Protein–protein interaction network

The protein–protein interaction (PPI) network was constructed based on all DEGs using the online tool STRING (https://string-db.org/) (32).Visualization was performed using Cytoscape software (version 3.9.0) (33). Significant gene clusters were identified using Minimal Common Oncology Data Elements (MCODE) and were scored accordingly (34).

### 2.4 Functional enrichment

The ClusterProfiler package of R was used to perform gene ontology (GO) enrichment analysis and Kyoto Encyclopedia of Genes and Genomes(KEGG) (35). The species restriction was set to *Homo sapiens*, and an “adjusted p -value (from the Benjamini–Hochberg method) of <0.05” was considered to indicate statistical significance. The GO terms included: biological process, cellular component, and molecular function.

### 2.5 Gene set enrichment analysis

Biological signaling pathways involved in genes were explored using gene set enrichment analysis software (version 4.2.2)(https://www.gsea-msigdb.org/gsea). Kyoto encyclopedia of genes and genomes enrichment data were screened based on net enrichment score, gene proportion, and p-value. A p-value of <0.05 and FDR q of <0.25 were considered to indicate significant enrichment.

### 2.6 Analysis of immune cell infiltration

Using the “CIBERSORT” package (36), we analyzed the abundance of 22 different immune cell subpopulations based on gene expression data analysis. Ten different immune cell subpopulations were identified using “immunedeconv” package (37).

### 2.7 Clinical samples

We collected testicular tissue from patients with azoospermia who presented to the Department of Urology at Hebei Medical University’s Second Hospital from February to November 2022 and underwent a testicular biopsy, all of whom were histopathologically and clinically diagnosed. The study protocol was approved by the Ethics Committee of Hebei Medical University’s Second Hospital, and written informed consent was obtained from each patient.

### 2.8 Cell lines and transfection

The TM4 mouse supporting cell line (Procell, China) was obtained and cultivated using a specific full TM4 culture (Procell, China). Cells were cultured under a humidified atmosphere with 95% air and 5% CO2. siRNA-DDX58 and siRNA-p65(GenePharma) were used to inhibit DDX58 and p65 expression; siRNA NC served as a control. According to the manufacturer’s guidelines, TM4 cells were incubated with Lipofectamine RNAiMAX(Thermo Fisher Scientific) and siRNA for 48 h. The siRNA sequences used in this study were as follows:

siRNA-DDX58 sense: 5′-GGUCUUCUUCGCUAACCAATT-3′
siRNA-DDX58 antisense: 5′-UUGGUUAGCGAAGAAGACCTT-3′
siRNA-p65 sense: 5′-GGAGUACCCUGAAGCUAUATT-3′
siRNA-p65 antisense: 5′-UAUAGCUUCAGGGUACUCCTT-3′

### 2.9 RNA extraction and reverse transcription quantitative polymerase chain reaction

Clinical testicular tissues and treated cells were lysed using QIAzol Lysis reagent (79306). According to the manufacturer’s instructions, total RNA was extracted using miRNeasy Mini Kit (217004; Qiagen). RNA yield was assessed using NanoDrop 2000. M-MLV First Strand Kit (Life Technologies) and random hexamer primers were used to synthesize cDNA from mRNA. mRNAs were subjected to reverse transcription quantitative polymerase chain reaction (RT-qPCR) using the primers listed in **Table 2** *via* Platinum SYBR Green qPCR SuperMix UDG Kit (Invitrogen) and ABI 7500 FAST System (Life Technologies). The relative transcript expression levels were calculated using 2−ΔΔCt formula and were normalized to those of GAPDH, as previously described (38).

**Table 2:** Primer sequences for RT-qPCR.

Gene name	Species	Sense (5′–3′)	Antisense (5′–3′)	Amplicon (bp)
DDX58	Human	TGCGAATCAGATCCCAGTGTA	TGCCTGTAACTCTATACCCATGT	83
GBP1	Human	AGGAGTTCCTTCAAAGATGTGGA	GCAACTGGACCCTGTCGTT	520
IFI16	Human	TAGAAGTGCCAGCGTAACTCC	TGATTGTGGTCAGTCGTCCAT	179
PARP9	Human	TCTGATGGGATTCAACGTGGA	TTCCTGGGCTGATAATTTCTGTG	188
IFI44	Human	GGTGGGCACTAATACAACTGG	CACACAGAATAAACGGCAGGTA	95
GBP2	Human	CATCCGAAAGTTCTTCCCCAA	CTCTAGGTGAGCAAGGTACTTCT	79
IFIT1	Human	AGAAGCAGGCAATCACAGAAAA	CTGAAACCGACCATAGTGGAAAT	112
IFI44L	Human	AGCCGTCAGGGATGTACTATAAC	AGGGAATCATTTGGCTCTGTAGA	116
SAMD9L	Human	ATTCCAAGCAACGGGATGTAG	AGTCTCGGTTTCCTATGAGAAGT	159
PLSCR1	Human	GGCATTTACAGACGCTGATAACT	AGGCACCAATCATTACAGCTTT	83
HERC6	Human	ATTTGGAGACAATCGCTCTGG	TGCGAAACTAGGCCATCAATTC	96
GAPDH	Human	TGTGGGCATCAATGGATTTGG	ACACCATGTATTCCGGGTCAAT	119
DDX58	Mouse	AATATGTGCCCCTACTGGTTGT	CGAAGAAGACCACTTTCCCTTT	107
P65	Mouse	AGGCTTCTGGGCCTTATGTG	TGCTTCTCTCGCCAGGAATAC	111
IL-6	Mouse	TAGTCCTTCCTACCCCAATTTCC	TTGGTCCTTAGCCACTCCTTC	76
IL-18	Mouse	GACTCTTGCGTCAACTTCAAGG	CAGGCTGTCTTTTGTCAACGA	169
IL-1β	Mouse	GCAACTGTTCCTGAACTCAACT	ATCTTTTGGGGTCCGTCAACT	89
GAPDH	Mouse	AGGTCGGTGTGAACGGATTTG	TGTAGACCATGTAGTTGAGGTCA	123

### 2.10 Western blot analysis

Western blot analysis was conducted as previously described (39). Proteins were extracted from cultured cells with RIPA lysis buffer. Equal amounts of protein samples were electro-transferred to polyvinylidene fluoride membranes (Millipore) and were analysed using SDS–PAGE. The membranes were then blocked for 2 h with 5% milk. Thereafter, the membranes were incubated overnight with primary antibodies at 4°C. The following primary antibodies were used: anti-DDX58 (1:500, 20566-1-AP), anti-NF-κB p65 (1:1000, 10745-1-AP), anti-IL-6 (1:1000, 21865-1-AP), anti-IL-18 (1:1000, 10663-1-AP), anti-IL-1β (1:1000, 26048-1-AP), and anti-β-actin (1:5000, 81115-1-RR). Then, the membranes were incubated with horseradish peroxidase-conjugated secondary antibodies (1:5000, Rockland) for 2 h at room temperature. Blots were obtained using Millipore’s ImmobilioTM Western (Millipore) and examined with enhanced chemiluminescence Fua-zon Fx (Vilber-Lourmat). Finally, gray scale values were calculated.

### 2.11 Morphology and histology

Human testicular tissues were embedded in paraffin after fixation with formalin. Hematoxylin and eosin staining was performed using 10 consecutive 4-μm thick sections. Using LAS V.4.4 (Leica), cross-sectional images were obtained under a microscope (Leica DM6000B, Switzerland).

### 2.12 Immunohistochemical staining and evaluation

In brief, immunohistochemistry staining was performed using 4-μm paraffin-embedded tissue cross-sections. These sections were deparaffinized with xylene, hydrated with ethanol, and blocked with 10% normal goat serum (710027, KPL, USA). Then, the sections were incubated overnight with primary antibodies (DDX58,1:200, 20566-1-AP) at 4°C. Subsequently, the sections were incubated with secondary antibodies (horseradish peroxidase-labelled rabbit IgG antibody, 021516, KPL, USA). Finally, coloration reaction was performed using DAB matrix kit. After completing the staining and washing, clarifying, and analysis of the sections (40).

### 2.13 MTT assay

The 3-(4,5-dimethylthiazol-2-yl)-2,5-diphenyltetrazolium bromide (MTT) colorimetric assay was used to detect cell viability. In brief, TM4 cells were plated on 96-well plates and incubated with different dosages of D-galactose (D-gal; 0–50 g/L) (HY-N0210, MedChemExpress) for 48 h. Subsequently, 20 μL of MTT reagent (5 mg/mL; Sigma Aldrich) was added to each well and incubated for 4–6 h. Then, using a microplate reader (Thermo Fisher, USA), absorbance was measured at 450 nm (41).

### 2.14 Flow cytometry analysis

D-gal or siRNA was incubated with TM4 cells for 48 h. After washing with phosphate-buffered saline, cells were fixed overnight at −20°C in 75% (v/v) ethanol under ice cold conditions. The cells were resuspended in phosphate-buffered saline and analysed using the DNA content quantitation assay (Cell Cycle; CA1510, Solarbio). In addition, changes in the levels of reactive oxygen species, mitochondrial membrane potential, and apoptosis in D-gal-treated cells were detected using the corresponding kits, including a Reactive Oxygen Species Assay Kit (CA1410, Solarbio) (42), Mitochondrial Membrane Potential Assay Kit with JC-1(M8650, Solarbio) (43), and Annexin V-FITC apoptosis detection kit (A005-3,7 Sea Pharmatech, Shanghai, China) (44). A flow cytometry system (BD LSR Fortessa, Indianapolis, NJ, USA) was used to analyse the results of these four tests.

### 2.15 RNA immunoprecipitation assay

As per the manufacturer’s instructions, TM4 cells were collected and used in RNA immunoprecipitation experiments along with DDX58 antibody (20566-1-AP), IgG, and Dynabeads TM Protein G Immunoprecipitation Kit (10007D, Thermo Fisher). RNA pulled down by antibodies was evaluated using NanoDrop2000. Using random hexamer primers and M-MLV First Strand Kit (Life Technologies), cDNA was synthesized and subjected to RT–qPCR for quantitative analysis (41).

### 2.16 Statistical analysis

Statistical data are represented as means ± standard error. Student’s t test was used to compare data between two groups. Using one-way analysis of variance, the significance of the mean value differences between groups was examined. A p-value of <0.05 was considered to indicate statistical significance. Statistical analyses were performed using GraphPad Prism 8 software.

## 3 Results

### 3.1 Transcriptomic profiles of OA and SCOS samples

**Figure 1:**
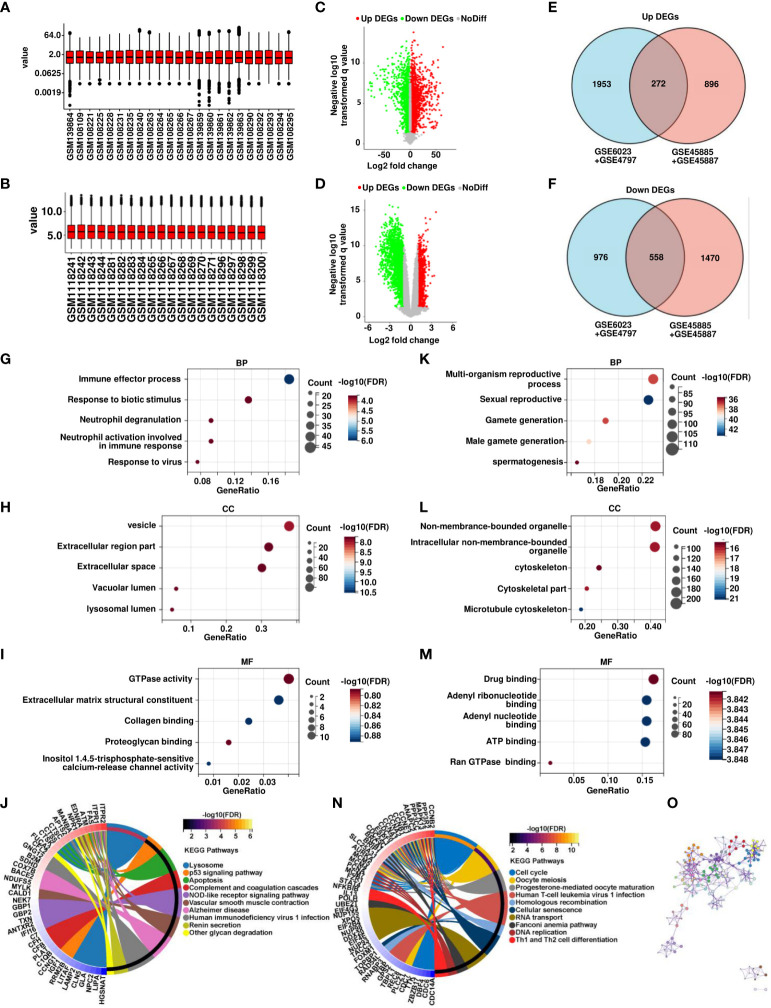
Analysis of transcriptomic profiles of OA and SCOS samples. **(A)** Expression data normalization in GSE6023 and GSE4797. **(B)** Expression data normalization in GSE45885 and GSE45887. **(C, D)** Volcano map of significant differential genes between SCOS and OA samples in GSE6023 and GSE4797 as well as GSE45885 and GSE45887. **(E, F)** Wayne plots of overlapping candidate genes were up- and downregulated in both combined datasets. Gene ontology (GO) analysis of (GK) biological process (BP), **(H, L)** cellular component (CC), **(I, M)** molecular function (MF), and **(J, N)** Kyoto encyclopedia of genes and genomes enrichment of up- and downregulated differential genes. **(O)** Construction of differential gene PPI networks based on STRING database.

Four datasets containing SCOS tissue sample data were obtained from the GEO database to search for differential genes in SCOS (**Table 1**). Samples from the same platform were merged, 4 normal spermatogenic samples and 7 SCOS samples in GSE45885, 4 normal spermatogenic samples and 5 SCOS samples in GSE45887 were combined into one dataset. 1 normal spermatogenic specimen and 5 SCOS samples in GSE6027 and 12 normal spermatogenic specimens and 5 SCOS samples in GSE4797 were integrated into another dataset, and the combined data were normalized, using the normalize Between Arrays method (**Figures 1A, B**). We identified 2225 upregulated genes and 1524 downregulated genes in the combined dataset of GSE6023 and GSE4797 in the SCOS sample (**Figure 1C**). In the combined dataset of GSE45885 and GSE45887, 1168 upregulated genes and 2028 downregulated genes were screened out in SCOS samples (**Figure 1D**)., Using the intersect of two datasets, we identified 272 upregulated and 558 downregulated DEGs (**Figures 1E, F**). We used GO and Kyoto encyclopedia of genes and genomes pathway analysis to investigate the biological function of DEGs. Highly expressed DEGs were mainly enriched in vesicles, extracellular regions, extracellular matrix structural constituents, and lysosomes as well as biological processes, such as immune effector process, response to biotic stimulus, vesicle, extracellular region part, GTPase activity, extracellular matrix structural constituent, Lysosome, p53 signaling pathway, and apoptosis pathway (**Figures 1G–J**). DEGs with low expression were mainly enriched in multi-organism biological processes, multi-organism reproductive process, sexual reproduction, non-membrane-bounded organelle, drug binding, adenyl ribonucleotide binding, cell cycle, and oocyte meiosis, as well as nonmembrane-bounded organelles (**Figures 1K–N**). Additionally, we constructed and visualized the PPI network using STRING after importing the DEGs into the network. These significantly differentially expressed genes are interrelated and constitute the main link in signal changes in SCOS (**Figure 1O**).

### 3.2 DDX58 is a vital regulator of SCOS development

**Figure 2:**
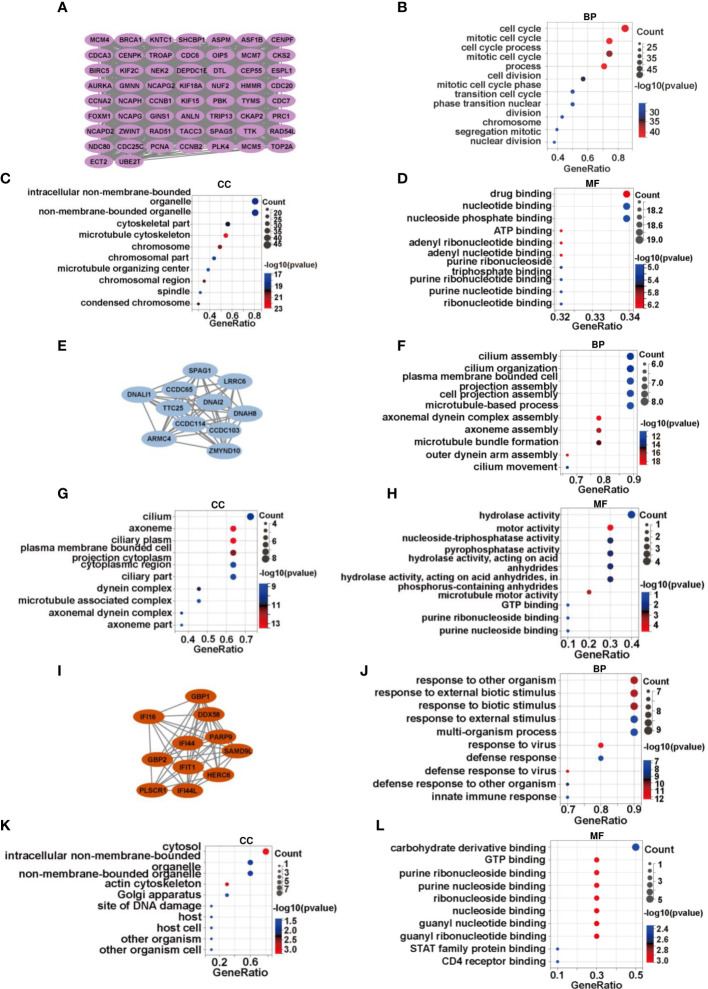
DDX58 is a vital regulator of SCOS development. **(A)**There are 58 genes in module 1, with a score of 51.236. Gene ontology analysis of genes in module 1 in terms of **(B)** biological process, **(C)** cellular component, and **(D)** molecular function. **(E)** There are 11 genes in module 2, with a score of 11. **(F–H)** Functional annotation of genes in module 2 using GO analysis. **(I)** There are 11 genes of module 3, with a score of 10.2. **(J–L)** GO analysis for gene functional annotation in module 3.

The MCODE plug-in was used to identify significant modules in PPI networks. The highest recognition rates were observed in three modules. GO enrichment analysis revealed that module 1 comprised 58 nodes and 1461 edges related to cell cycle, mitotic cell cycle, and other processes (**Figures 2A–D**). The 11 nodes and 55 edges in module 2 were mostly associated with cilium assembly, cilium organization, and plasma membrane bound cells (**Figures 2E–H**), whereas biological processes such as response to other organism and biotic stimulus were mostly enriched in the 11 nodes and 51 edges of module 3 (**Figures 2I–L**). The genes in modules 1 and 2 were downregulated, whereas the related genes, linked to spermatogonia, spermatocytes, sperm, and other related components, were mainly enriched in the testicular deletion part of SCOS. We decided to test HUB genes in module 3 because all genes in this module were upregulated and may reflect the disease status of SCOS testis, especially Sertoli cells. Based on the degree in Cytoscape, DDX58 was identified as the HUB gene (**Table 3**).

**Table 3:** Topological analysis results by degree—The third Protein-protein interaction networks in MCODE analysis.

Gene names	Annotation	Degree
DDX58	DExD/H-box helicase 58	18
GBP1	Guanylate binding protein 1	13
IFI16	Interferon gamma inducible protein 16	13
PARP9	Poly(ADP-ribose) polymerase family member 9	13
IFI44	Interferon induced protein 44	12
GBP2	Guanylate binding protein 2	11
IFIT1	Interferon induced protein with tetratricopeptide repeats 1	11
IFI44L	Interferon induced protein 44 like	10
SAMD9L	Sterile alpha motif domain-containing protein 9-like	10
PLSCR1	Phospholipid scramblase 1	9
HERC6	HECT and RLD domain containing E3 ubiquitin protein ligase family member 6	9

### 3.3 Increased DDX58 expression in the testicular tissue of patients with SCOS

**Figure 3:**
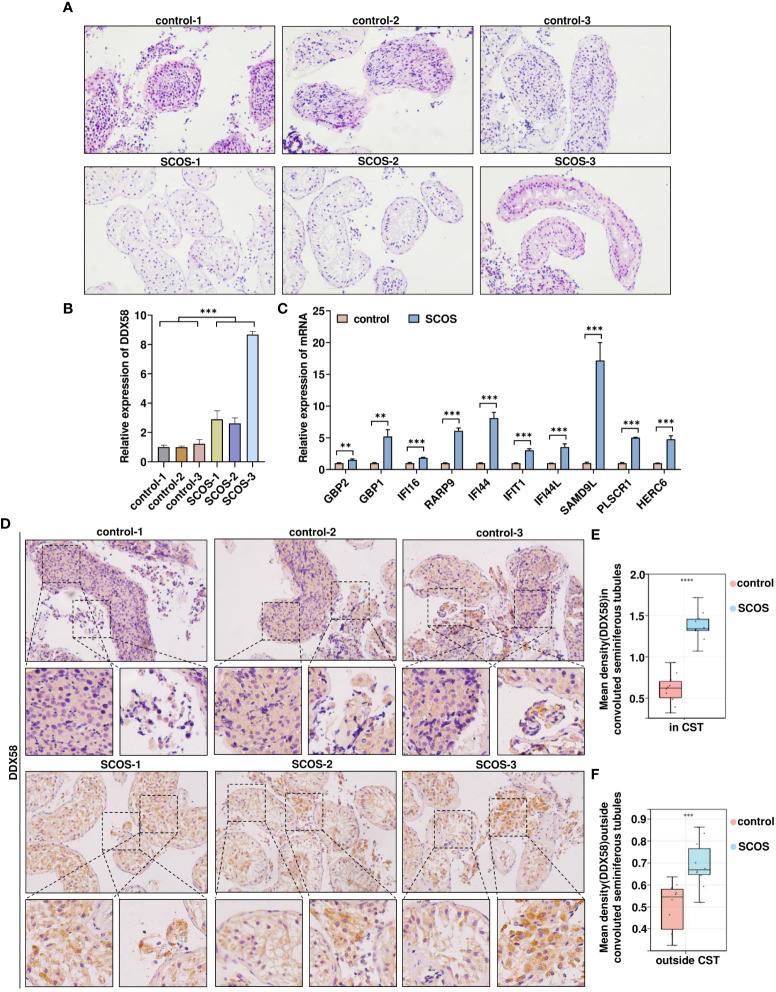
Elevated DDX58 expression in testicular tissue of patients with SCOS. **(A)** SCOS testis and spermatogenic normal testis, HE of hematoxylin and eosin. **(B)** RT-qPCR was used to detect DDX58 mRNA expression in testicular tissue. ***p < 0.001 *vs*. the control group. **(C)** RT-qPCR was used to detect mRNA expression of other 10 genes belonging to module 3 in testicular tissue. **p < 0.01, ***p < 0.001 *vs*. the control group. **(D)** Immunohistochemical staining of DDX58 in testes with OA and SCOS. Scale bar: 50 μm. Original magnification: ×200(n= 10). **(E)** Average DDX58 staining intensity in CST (n= 10). (CST, convoluted seminiferous tubule) ***p < 0.001 *vs*. the control group. **(F)** Average DDX58 staining intensity outside convoluted seminiferous tubules (n= 10). ****p < 0.0001 *vs*. the control group.

To investigate the relationship between DDX58 and SCOS, testicular biopsies were collected from patients with SCOS and those with OA; moreover, biopsies collected from those with normal spermatogenesis were used as controls. Hematoxylin and eosin staining indicated that spermatogenic cells and sperms in the control group were located in the spermatogenic tubules of the testis, indicating normal spermatogenic function. However, only Sertoli cells were visible in the tubules of SCOS tissue (**Figure 3A**). Further, DDX58 mRNA expression in SCOS testicular tissue, was considerably higher than that in the control tissue (**Figure 3B**). Additionally, SCOS testis tissue exhibited high mRNA expression for other 10 genes in module 3 (**Figure 3C**). Immunohistochemical staining was performed to further confirm DDX58 expression in SCOS tissues. As shown in **Figure 3D**, DDX58 protein expression in SCOS tissue was significantly increased compared with that in the control tissue. In particular, DDX58 expression was higher in SCOS testicular tissue, Sertoli cells in the convoluted seminiferous tubule, and cells outside the convoluted seminiferous tubule (**Figures 3E, F**). These findings demonstrate that DDX58 expression is upregulated in SCOS testicular tissue, particularly in Sertoli cells.

### 3.4 DDX58 expression is positively correlated with the intratesticular inflammatory response of SCOS

**Figure 4:**
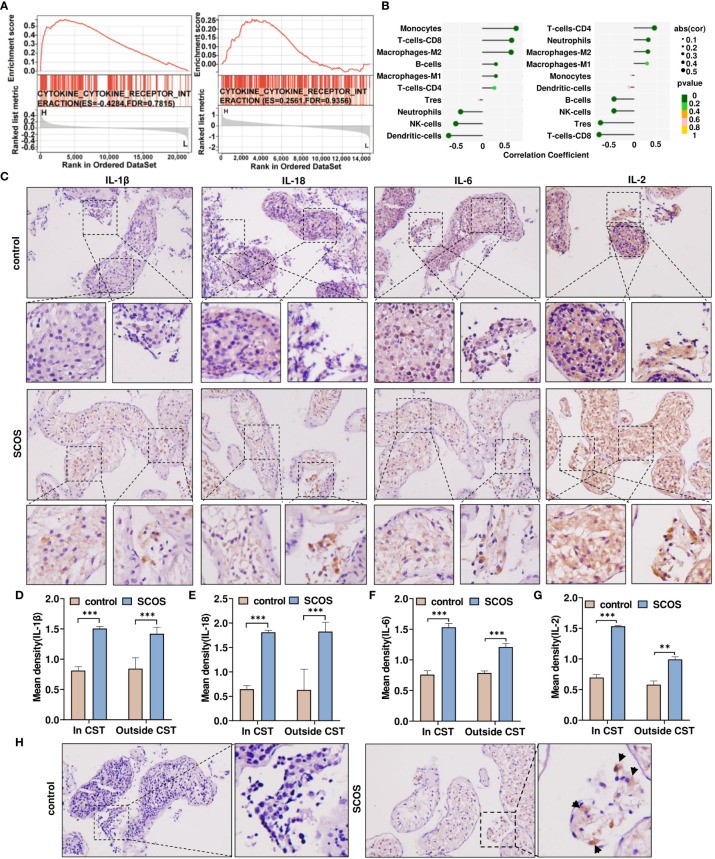
DDX58 expression is positively correlated with the intratesticular inflammatory response of SCOS. **(A)** GSEA results of cytokine-cytokine receptor interaction pathways in two datasets were compared between the samples with high and low DDX56 expression. **(B)** Lollipop plots showing the correlation between the expression of DDX58 and different TIIC ratios analyzed using quanTIseq and CIBERSORT. **(C)** Immunohistochemical staining of IL-1β, IL-18, IL-6, and IL-2 in testes with OA and SCOS. Scale bar: 50 μm. Original magnification: ×200 (n= 10). **(D–G)** Average IL-1β, IL-18, IL-6, and IL-2 staining intensities in and outside the CST. **p < 0.01, ***p < 0.001 *vs*. the control group. **(H)** Results of macrophage immunohistochemical staining in SCOS and OA testis tissues marked with F4/80 antibodies. Scale bar: 50 μm. Magnification: ×200.

We performed a gene set enrichment analysis based on DDX58 expression and found that cytokine–cytokine receptor interaction was mainly enriched with high expression of DDX58 (**Figure 4A**). In addition, correlation analysis revealed that DDX58 had high confidence in regulating inflammatory cytokine expression and immune cell infiltration (**Figure 4B**). To further examine inflammatory cytokine expression in testicular tissue, we performed immunohistochemical staining. As shown in **Figures 4C–G**, the expression of inflammatory cytokines, such as IL-1β, IL-6, IL-18, and IL-2 was significantly increased in Sertoli and extratubular cells in the convoluted seminiferous tubules of SCOS testes, indicating the inflammatory seminiferous environment of SCOS testes, which may be related to DDX58. Next, we found F4/80-positive macrophage infiltrates between the seminiferous tubules of SCOS testis (**Figure 4H**). These results suggest the existence of a significant inflammatory response in the testicular tissue of SCOS, which may be positively correlated with DDX58 expression.

### 3.5 D-gal- induced cell growth arrest and chronic inflammatory response

Studies have reported that D-gal can induce cellular oxidative stress, mitochondrial damage, inflammation, and tissue damage (45–47).

**Figure 5:**
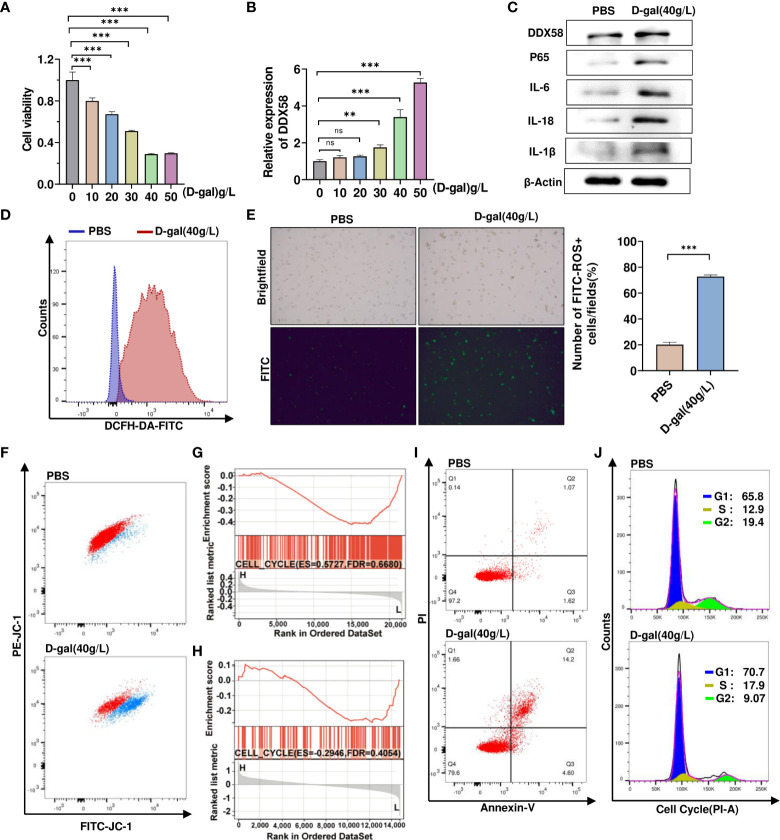
D-gal- induced cell growth arrest and chronic inflammatory response. **(A)** CCK8 analyzes TM4 cell viability treatment with varied D-galactose concentrations. *** p < 0.001; ns indicates no statistical significance. **(B)** RT–qPCR was used to detect mRNA expression of DDX58 after treatment with various doses of D-galactose. ** p < 0.01, *** p < 0.001; ns indicates no statistical significance. **(C)** Western blot analysis was used to detect the levels of protein expression of DDX58, p65, and inflammatory factors in control and D-gal-treated cells. **(D, E)** Flow cytometry and immunofluorescence were used to detect the levels of ROS in cells treated with 40 g/L D-gal. ***p<0.001. **(F)** Flow cytometry was used to detect the proportion of cells with low mitochondrial membrane potential after D-gal treatment. **(G, H)** GSEA results for cell cycle pathways in two datasets were compared between the samples with high and low DDX58 expression. **(I)** Using flow cytometry, the proportion of apoptotic cells were shown in control (1.07%) and D-gal-treated cells (14.2%). **(J)** Following D-gal treatment, flow cytometry was used to detect changes in cell cycle.

We first treated TM4 cells with different concentrations of D-gal and then performed cell viability assays. D-gal significantly reduced cell viability in a dose-dependent manner (**Figure 5A**). Next, we observed a gradual increase in DDX58 mRNA expression with increasing D-gal concentrations (**Figure 5B**). We found that 40g/L D-gal considerably increased DDX58 mRNA expression and significantly inhibited cell viability. Next, we used 40 g/L D-gal to stimulate TM4 cells. As expected, D-gal-treated TM4 cells significantly increased DDX58 and inflammatory cytokine expression (**Figure 5C**). To explore whether D-gal induced TM4 cellular oxidative stress, a FITC-labeled ROS probe was used to detect ROS levels; flow cytometry and immunofluorescence analysis revealed that 40g/L D-gal considerably increased ROS production in the mitochondria (**Figures 5D, E**). In addition, D-gal treatment significantly decreased mitochondrial membrane potential (**Figure 5F**), thereby promoting oxidative stress in cells and exacerbating cell damage. Subsequently, a gene set enrichment analysis showed that DDX58 was significantly associated with cell cycle pathways (**Figure 5G, H**). Moreover, flow cytometry analysis revealed that D-gal treatment significantly promoted apoptosis and decreased the number of G2 phase cells (**Figures 5I, J**). These results suggest that D-gal regulates chronic inflammatory responses and growth arrest in these cells.

### 3.6 DDX58 is involved in D-gal-induced cell growth arrest and cellular inflammatory responses

**Figure 6:**
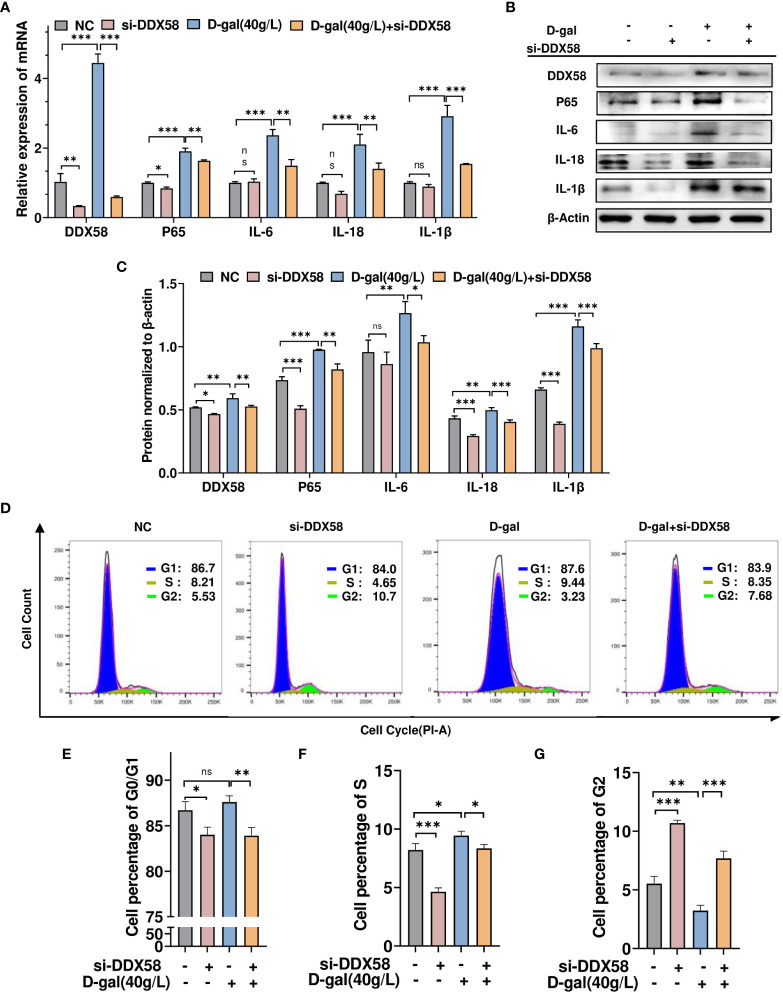
DDX58 is involved in D-gal-induced cell growth arrest and cellular inflammatory responses. **(A)** RT–qPCR and **(B)** Western blot analysis was used to detect DDX58, p65, IL-1β, IL-18, and IL-6 expression in cells transfected with siRNA-DDX58 and D-gal alone or in combination. **(C)** The panel shows the grayscale analysis. **(D)** Flow cytometry analysis of the cell cycle in the four cell groups in **(A)** showed the percentage of cells at various phases of division. These graphs indicate the changes in **(E)** G0/G1, **(F)** S, and **(G)** G2 phases in different cell groups. * p < 0.05, ** p < 0.01, *** p < 0.001; ns indicates no statistical significance.

To investigate whether DDX58 mediates the inflammatory response and cell growth arrest caused by D-gal-induced cell damage.TM4 cells were transfected with siRNA-DDX58 and treated with or without D-gal. Then, RT-qPCR was used to determine inflammatory cytokine levels. As shown in **Figure 6A**, D-gal treatment promoted p65, IL-6, IL-18, and IL-1β expression. However, this effect was partially opposed by simultaneous transfection with siRNA-DDX58. This finding was consistent with Western blot result (**Figures 6B, C**). Subsequently, the treated cells were analyzed using flow cytometry. Compared with the control group, the number of G2 phase cells significantly decreased and that of G1 and S phase cells increased in TM4 cells treated with D-gal. However, the combination of DDX58 knockdown and D-gal treatment, blocked D-gal-induced cell growth arrest and promoted G1/S phase cell passage to G2 phase (**Figures 6D–G**). These results suggest that DDX58 is involved in the chronic inflammatory responses and growth arrest induced by D-gal in these cells.

### 3.7 DDX58 promotes p65 expression by stabilizing p65 mRNA

DDX58 reportedly promotes downstream gene expression by recognizing and binding to double or single-stranded RNA (18, 20).

**Figure 7:**
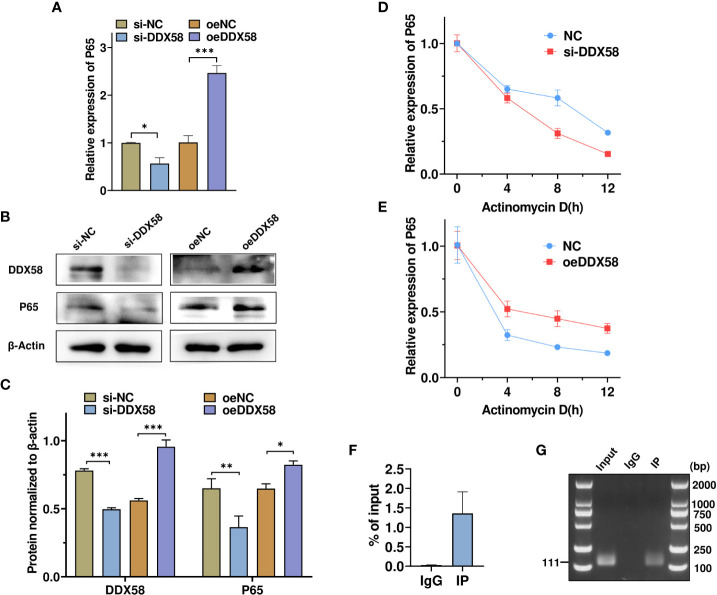
DDX58 promotes p65 expression by stabilizing p65 mRNA. **(A)** RT–qPCR was used to detect changes in p65 mRNA expression after DDX58 overexpression and DDX58 knockdown. * p < 0.05, *** p < 0.001. *vs*. the corresponding control. **(B, C)** Western blot and grayscale analyses revealed the expression of DDX58 and p65 protein in **(A)**. **(D, E)** Cells were treated as described in **(A)** **p < 0.01. and were then exposed to Actinomycin D for 0, 4, 8, and 12 h. p65 mRNA expression was detected using RT–qPCR. * p < 0.05. *vs*. the corresponding control. **(F)** RNA-binding protein immunoprecipitation PCR was used to analyze the interaction between DDX58 protein and p65 mRNA. **(G)** Agarose electrophoresis was used to detect the PCR products from **(F)**.

We noted that the knockdown of DDX58, an RNA -binding protein, decreased p65 mRNA and protein expression (**Figures 6A–C**). To confirm whether DDX58 promoted p65 expression, we overexpressed or knocked down DDX58 and examined p65 mRNA expression using RT-qPCR. As shown in **Figures 7A–C**, DDX58 knockdown reduced p65 mRNA and protein expression, whereas its overexpression showed the opposite effect. To investigate the mechanism by which DDX58 regulates p65 expression, TM4 cells were transfected as described previously and then treated with Actinomycin D to inhibit gene transcription. Subsequently, p65 mRNA expression at different time points was measured using RT-qPCR. As shown in **Figures 7D, E**, DDX58 overexpression increased and DDX58 knockdown reduced p65 mRNA expression. RNA immunoprecipitation using DDX58 antibodies confirmed that DDX58 antibodies interact with p65 mRNA (**Figure 7F**). Moreover, based on agarose gel electrophoresis, p65 mRNA can be pulled down by DDX58 antibodies (**Figure 7G**). These results demonstrate that DDX58 promotes p65 expression by stabilizing mRNA.

### 3.8 p65 mediates D-gal-induced testicular cell growth and inflammation in SCOS

**Figure 8:**
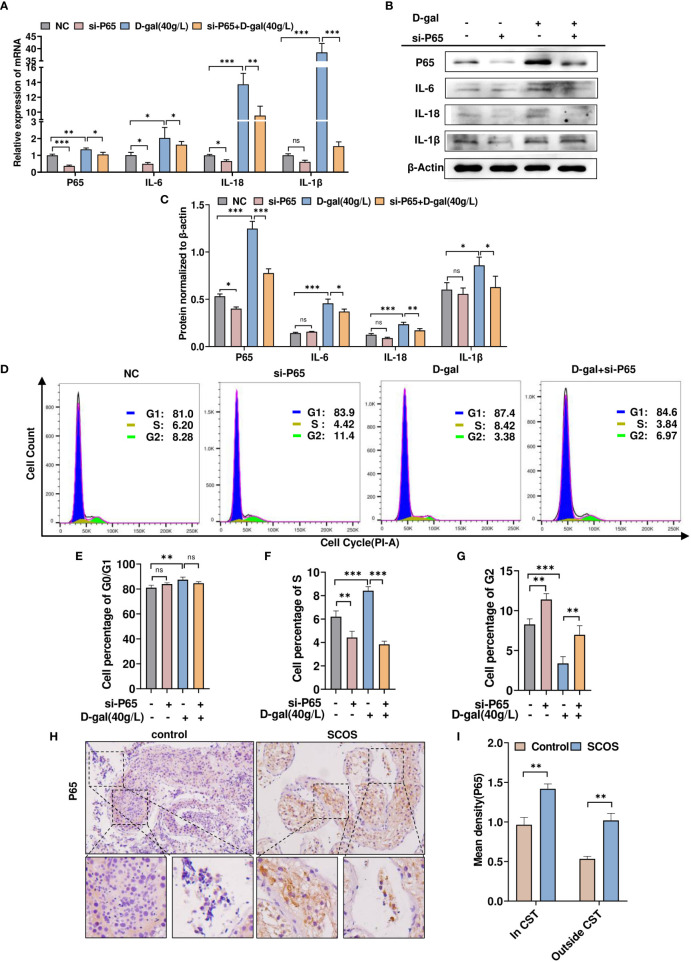
p65 mediates D-gal-induced testicular cell growth and inflammation in SCOS. **(A, B)** RT–qPCR and Western blot analysis were used to detect p65, IL-1β, IL-18, and IL-6 expression in cells transfected with siRNA-p65 and D-gal alone or in combination. **(C)** The panel shows the grayscale analysis of data presented in **(B)**. **(D)** Flow cytometry analysis of the cell cycle in the four cell groups in **(A)** showed the percentage of cells at various phases of division. **(E–G)** These graphs indicate the changes in G0/G1, S, and G2 phases in different cell groups. ** p < 0.01, *** p < 0.001; ns indicates no statistical significance. **(H)** Immunohistochemical staining revealed p65 expression in testes with normal spermatogenesis and those with SCOS. **(I)** Average p65 staining intensity in and outside CST. **p < 0.01 *vs*. the control group. Scale bar: 50 μm. Original magnification: ×200.

To determine whether DDX58-mediated D-gal-induced inflammatory responses and cell growth arrest are regulated by P65, TM4 cells were transfected with siRNA-p65 and treated with D-gal simultaneously. RT-qPCR revealed that D-gal treatment significantly increased IL-18, IL-1β, and IL-6 expression, whereas p65 knockdown counteracted the effect of D-gal treatment (**Figure 8A**). Similar results were obtained using Western blot (**Figures 8B, C**). Next, we examined cell cycle changes *via* flow cytometry and found that p65 knockdown increased the number of G2 phase cells; however, after D-gal treatment, p65 knockdown significantly increased G2 phase cells and promoted their growth (**Figures 8D–G**). Subsequently, we detected p65 expression in SCOS testicular tissue; in particular, high p65 expression was observed in the seminiferous tubules and interstitial tissue (**Figures 8H, I**). Altogether, these results support that p65 mediates D-gal-induced inflammatory responses and cell growth in SCOS.

## 4 Discussion

SCOS is a serious pathological type of male infertility, and no effective treatment is available for this condition. Numerous medical practitioners and scholars have explored the cause of this disease and revealed primary genetic defects. AZFa microdeletion has been associated with male infertility, and AZFa deletion cases are detected in the SCOS population (9, 48, 49). Studies have reported that mutations in single genes encoding proteins such as ETV5, PIWIL2, and CULB4 are associated with SCOS (12, 50, 51). In addition, SNP microarray analysis of patients with SCOS by Sharma et al. indicated that variants and polymorphisms in some genes play a vital role in the progression of SCOS (52). Moreover, drug usage or varicocele may impair spermatogenic cells in animal models and individual patients (53–55). However, its pathogenesis remains unclear.

It has been hypothesized that inflammatory responses within the testis and the resulting testicular injury play a role in the pathogenesis of SCOS. For example, the seminiferous epithelium gradually deteriorates due to mumps-related orchitis, and tubules of Sertoli cell-only syndrome could be occasionally involved (56). Diethylstilbestrol-induced epididymitis leads to granulomatous orchitis, resulting in pathological conditions similar to SCOS in some tubules (53). Carolyn et al. observed localized SCOS and spermatogenic cell damage in patients with canine spontaneous orchitis (57). Moreover, according to Chen et al, the expression of inflammation-related genes was increased in SCOS tissues (58). Accordingly, we verified the abnormal elevation in the levels of inflammatory factors in the testes of patients with SCOS.

The development of SCOS may be multifactorial. Nistal has stated that different types of Sertoli cells, including mature, dysplastic, degenerated, and normal cells, are formed in SCOS due to different causes (6). Dorien et al. examined the distribution of extracellular matrix in the testes of patients with SCOS and reported abnormal expression and distribution of extracellular matrix-related proteins (59). Although fibrosis is most often observed in the testicular interstitium, hyaline degeneration can also occur due to the presence of specialized epithelial cells in the seminiferous epithelium. Further, Maria et al. examined tight structure-related proteins in Sertoli cells of patients with SCOS and revealed abnormal distribution of proteins related to the blood-testis barrier (60). Immature cells, aging, degeneration, inflammation, hyalinization, or fibrotic changes in the seminiferous epithelium could contribute to the development of SCOS. DDX58 may be involved in these changes or some other unknown pathological changes, thereby contributing to the pathogenesis of SCOS. Although research on animal models has already been performed (61, 62), their use is still limited because of the unknown etiology of SCOS and the need for detailed analysis. To further improve our data analysis, we need to expand the sample size of this study.

DDX58, which encodes DExD/H -box RNA helicase with a caspase recruitment domain, is a key regulator of dsRNA-induced signaling (18, 63). Recently, numerous studies have revealed that DDX58 plays a vital role in nonviral systems: influencing tumor prognosis (64), participating in the regulation of cell proliferation and differentiation (21), aging (65, 66), and autophagy (67). Interestingly, our results not only demonstrated that DDX58 expression was abnormal in the Sertoli cells of the SCOS testis’s seminiferous tubules, but also increased DDX58 expression in the Leydig cells outside the seminiferous tubules as compared to the normal spermatogenic group (**Figure 3F**). In SCOS testicular tissue, the proliferation of Leydig cells in the interstitium of the testes to form micronodules is a typical feature of SCOS (68). The regulation of granulocyte proliferation and differentiation is significantly influenced by DDX58 (21). DDX58 may be involved in the process of Leydig cell proliferation. Additionally, Zhou et al. found that DDX58 is involved in the progression of renal fibrosis (69). Similarly, Leydig cells and Sertoli cells are essential members of the testes’ normal hormone production (70, 71). The DDX58-mediated inflammatory response involves unknown changes in spermatogenic epithelial and Leydig cells, and the possible effects on hormone secretion are worth exploring. To the best of our knowledge, this is the first study presenting the abnormal expression of DDX58 in SCOS testes. Moreover, we found significantly higher DDX58 expression in Sertoli cells than in normal seminiferous testes. In addition, the D-gal-induced TM4 cell injury model increased significantly its expression. These results highlight the association between DDX58 and Sertoli cell dysfunction. We further revealed that DDX58 regulated cell proliferation and promoted inflammatory responses in Sertoli cells by activating p65. Mechanistically, DDX58 can not only detect exogenous RNA but also recognize other nucleic acid sequences produced by the cell. Marco et al. found that DDX58 plays a bridging role in signaling communication between mitochondria and nucleoplasm (20). We demonstrated that DDX58 could bind p65 mRNA and promote its stability, thereby increasing its expression and promoting inflammatory response (72, 73). In contrast to previous studies, we found novel forms of interaction between DDX58 and p65.

In conclusion, this study examined the role of DDX58 in SCOS. We found that DDX58 acts as an RNA-binding protein, stabilizes p65 mRNA, and promotes its expression, which in turn activates the release of proinflammatory factors and arrests the growth of Sertoli cells. Thus, wouldn’t inhibition of DDX58 induced inflammatory injury represent a possible therapeutic strategy for SCOS? There is no discussion on this possibility but this could be worthwhile.

## Data availability statement

The original contributions presented in the study are included in the article/supplementary material. Further inquiries can be directed to the corresponding authors.

## Ethics statement

The studies involving human participants were reviewed and approved by Medical Research Ethics Committee of the Second Hospital of Hebei Medical University. The patients/participants provided their written informed consent to participate in this study.

## Author contributions

HS and ZY performed the experimental design and conception. HS, ZT, CX, ZW and HuW acquired the data. HS, ZH, YZ, HoW and XC analyzed and interpreted the data. HS, ZY, CQ and YW wrote, reviewed, and/or revised the manuscript. All authors contributed to the article and approved the submitted version.
